# Vaccine hurdles for children in war-torn Somalia: the result of post-campaign coverage survey 2025

**DOI:** 10.1186/s13031-025-00727-4

**Published:** 2026-01-20

**Authors:** Abdulwahab M. Salad, Mohamed A. Gedi, Abdirazak A. Mire, Hodan A. Duale, Abu Obeida Eltayeb, Dahir A. Ali, Mustafa J. Awil, Mohamed M. Derow, Abdi Gele

**Affiliations:** 1 Somali Institute for Health Research, Mogadishu, Somalia; 2 World Health Organization, Mogadishu, Somalia; 3 Ministry of Health, Immunization program, Mogadishu, Somalia; 4 Simad University, Mogadishu, Somalia; 5 Norwegian Institute of Public Health, Oslo, Norway; 6 United Nations Children’s Fund (UNICEF), Somalia Office, Mogadishu, Somalia

## Abstract

**Background:**

The measles vaccine represents one of the most efficacious means of safeguarding pediatric populations against the measles virus. Empirical evidence consistently demonstrates that over 99% of recipients who complete the two-dose regimen achieve immunity to the disease. Despite this high efficacy, suboptimal vaccine coverage persists as a significant impediment to the containment and eventual eradication of measles. To augment immunization coverage, routine vaccination programs are often supplemented by second-dose opportunities administered through Supplemental Immunization Activities (SIAs), particularly in countries with a high measles burden. This study aims to evaluate the measles vaccination coverage attained after the nationwide SIAs in May 2025 as well as vaccination coverage prior to SIAs.

**Methods:**

This study was conducted from May 26th to June 5th, 2025. A two-stage cluster sampling design was utilized, where 938 clusters were randomly selected from a national sampling frame of 6,936. This sampling frame covered all accessible districts, villages, and nomadic areas throughout the country. The sample size was calculated using the WHO-2018 manual, resulting in the selection of 134 clusters and 1,780 households from each state, culminating in 12,832 interviews overall. A total of 355 interviewers, equipped with the KoboCollect app for digital data collection, conducted surveys. We calculated vaccine coverage during supplemental immunization activities (SIAs) and prior to SIAs (which means routine immunization). To calculate the national coverage, a survey weighting methodology was applied. Specific procedures were used to determine cluster and household weights, following the formula outlined in the WHO Vaccination Coverage Cluster Survey 2018. Both descriptive and inferential statistics were applied. Descriptive statistics, including frequency counts and proportions, enabled us to summarize the general attributes of the sample. Conversely, inferential statistics were used to estimate national vaccine coverage, featuring point estimates and confidence intervals (Wilson 95% Confidence Intervals), with the application of survey weights. Ethical approval was obtained from the Somali National University Ethical Committee.

**Results:**

Of 17,700 children across seven states, 46.5% were girls and 53.5% were boys. Most children (56.2%) lived in urban areas, followed by 25.8% in rural areas and 14.2% in nomadic areas. Additionally, 3.76% of children lived in Internally Displaced Persons (IDP) camps. Weighted coverage estimates indicate that 73% (CI: 68.14–77.3) of children in Somalia received the measles vaccine during SIAs. Regarding the regional variation of vaccine coverage, the highest coverage was observed in Somaliland (87.4%, CI: 81,7–91.3) and Puntland (87.1%, CI: 82.7–90.5), while the lowest were found in Hirshabelle (48%, CI: 32.6–63.8) and Southwest (50%, CI: 40.4–59.7) states. In terms of geographic settlements, the nomadic population had the lowest measles vaccine coverage during SIAs (70.9%, CI: 57.8–81.3), and had the highest zero-dose children of 15.4% (CI: 8.9–25.2) compared to urban and rural populations. The national measles coverage prior to SIA was found to be 78,6% (CI: 74.6–82.09). The SIAs achieved a national coverage rate of 39.8% among children who had previously received no doses (zero-dose children) and 82% among those who had received at least one dose prior. The predominant reason for children not being vaccinated during the SIAs was a lack of awareness about the vaccination campaign.

**Discussion:**

The lowest national vaccine coverage and the highest proportion of zero-dose children were predominantly found in the Hirshabelle and Southwest states and among nomadic communities. This difference in vaccine coverage among states and communities is concerning and suggests the need for targeted intervention to address these gaps.

## Background of the problem

Infectious diseases are a major cause of morbidity and mortality in children globally, with vaccination being one of the most important and cost-effective public health interventions available to combat infectious diseases among children [1]. It is estimated that 25% of the under-five mortality is due to vaccine-preventable diseases, yet an estimated 19.4 million children around the world remain un- or under-immunized [1]. The World Health Assembly, supported by countries including Somalia, has endorsed the Immunization Agenda 2030 to ensure global vaccine access, aiming to save over 50 million lives and improve health universally by maintaining immunization gains and reaching all individuals without exception [2]. In May 1974, the World Health Organization (WHO) officially launched a global immunization program known as the Expanded Program on Immunization (EPI), to protect all the children of the world against six vaccine-preventable diseases [3]. Vaccination programs are highly complex, particularly as they shift beyond ‘business as usual’ approaches to reaching all children by overcoming deep-rooted obstacles that hampered the universal immunization coverage. Improving the performance of programs requires a clear understanding of the bottlenecks to progress and strategies to address them, which implies that vaccine programs must be rigorously evaluated to learn and improve [4].

Moreover, armed conflicts often result in dramatic reductions in vaccine coverage, increasing the risk of outbreaks of diseases that vaccines can prevent [5]. Research spanning 16 countries affected by conflict revealed that, although these nations represent only 12% of the world’s population, they were responsible for 67% of global polio cases and 39% of measles cases from 2010 to 2015 [6]. Additionally, 14 out of these 16 nations reported DTP3 (diphtheria, tetanus toxoid, and pertussis) vaccination rates below the worldwide average of 85%, with significant declines noted following the emergence of conflict [6]. These observations concur with international predictions that indicate more than two-thirds of children who have not been vaccinated reside in areas impacted by conflict, highlighting a clear link between the interruptions caused by warfare and failures in maintaining immunization efforts [7].

Somalia has faced persistent armed conflict for several decades, which has significantly impacted its health indicators. The intensity and frequencies of conflicts are higher in South-central states compared to more stable Northern estates of Puntland and Somaliland. The ongoing instability in parts of the country have disrupted essential healthcare services and infrastructure, making it challenging for citizens to access necessary healthcare [8]. Vaccination programs have often been interrupted, leading to lower immunization rates and increased vulnerability to outbreaks of diseases such as measles and polio [9]. The displacement of people due to conflict further strains the fragile healthcare system, as displaced populations often lack access to clean water and adequate sanitation, heightening the risk of communicable diseases. The cumulative effect of these factors has led to poor health outcomes, with Somalia recording some of the highest infant and maternal mortality rates globally [8].

With the support of WHO and UNICEF, the EPI started in Somalia in 1978, using mobile and outreach services strategy. An evaluation of the program in 1985 showed that the strategy achieved very low immunization coverage. Between 1985 and 1988, a strategy of mass immunization campaign was implemented in major towns in Somalia. The operation resulted in 75% vaccine coverage of children in towns. However, this could not be sustained, as immunization coverage rapidly declined at the onset of the civil war in 1988 [3, 10]. The civil war devastated the health infrastructure and dispersed health workers hence completely reversed the modest gains of the program. The trend of low childhood immunization coverage was evident during the baseline survey conducted in Nov 2017, in which the immunization coverage in 9 districts was assessed for four basic vaccines, namely BCG (57%), Measles (47%), Polio (14%), and Pentavalent (28%) [5]. Further, a recent Somalia health demographic survey showed a poor coverage of routine vaccinations in Somalia, with only 10.7% of children aged 12–23 months being fully vaccinated [8]. This finding stands in stark contrast to the global trend observed in 2015, when approximately 85% of children worldwide received at least one dose of the measles vaccine by their first birthday through routine health services [11]. The 2023 Post Campaign Coverage Survey (PCCS) which was conducted in six states in Somalia (except Somaliland), showed lower coverage than administrator vaccination, and much lower coverage among measles-zero dose children vs. those previously vaccinated, suggesting that the SIAs was not that successful in reaching communities that get missed by routine immunization services [12]. Moreover, the large number of suspected measles cases in 2022, totaling 14,847 by epidemiological week 42, along with a high Measles IgM positive rate of 60% in Somalia, strongly indicate that children in the region have low immunity against measles. These figures suggest that a significant proportion of the child population is susceptible to the disease, highlighting the urgent need for improved vaccination coverage [8].

The routine immunization coverage for children is often enhanced by supplementing it with a second dose opportunity for measles vaccine through supplemental immunization activities (SIAs) in high measles burden countries including Somalia. In May 2025, Somalia successfully executed the Measles Containing Vaccine (MCV) Supplemental Immunization Activities (SIAs) campaign, with the objective of vaccinating all children aged 6 to 59 months. The campaign was executed in two distinct phases. Phase 1 was conducted from May 18th to May 22nd, 2025, targeting five South-Central states. Phase 2 occurred in the Puntland and Somaliland from May 25th to May 29th, 2025. The World Health Organization (WHO) Somalia Immunization Technical Advisory Group recommended that Somalia undertake a post-campaign coverage estimation survey in alignment with WHO survey guidelines. The PCCS-2022 results were the last survey that was documented, and it covered only a portion of the country [12]. The current PCCS is, therefore, the first of its kind to present the SIAs coverage across all states in Somalia, with the exception of 15 districts controlled by non-state actors, which were not reached by the SIAs.

The objective of the survey was to estimate the measles vaccine coverage achieved following the nationwide SIAs conducted in May 2025, and to analyze the heterogeneity of measles vaccine coverage across the various states in Somalia. Particular emphasis was placed on determining whether the SIAs effectively identified and vaccinated children who had been missed by routine immunization services.

## Methodology

### Study area

Somalia is a federal state consisting of six member states and Somaliland: Jubbaland, South-West, Benadir Administration, Hirshabelle, Galmudug, and Puntland. There is strong variability in security and accessibility of services across the states in the country, influenced by the frequency of conflict and the presence of government control in each state. The conflict between the government and non-state actors is particularly intense in the south-central part of the country, especially in the Hirshabelle and South-West states [13]. Additionally, a large part of Jubbaland state and several areas in Galmudug state are controlled by non-state actors, where conflict occurs sporadically. Conversely, Puntland state and Somaliland enjoy relative peace [13]. Aside from a minor presence of non-state actors in the far eastern mountainous areas of Puntland, both administrations have comparatively strong governance and security apparatus [13].

### Survey setting

The survey was conducted from May 26th to May 30th across five Federal Member States in Somalia; Galmudug, Hirshabelle, South-West, Jubbaland, and the Benadir administration, while the survey was conducted from 1 st to 5 st of June in Puntland state as well as Somaliland. The survey encompassed all accessible districts that participated in the SIAs in May 2025. Special attention was directed toward including remote and hard-to-reach areas, such as nomadic populations and underserved rural communities. The target population were children aged 6 to 59 months at the time of the SIAs. We used a sampling frame provided by the WHO. The sampling frame utilized for surveys in Somalia is grounded in the Population Estimation Survey of 2013 [14]. It is regularly updated to account for factors such as population growth and urbanization. The frame comprised a comprehensive list of clusters throughout the country, excluding 15 inaccessible districts located in four south-central states: Galmudug [5], Jubbaland [5], South-West [3], and Hirshabelle [2], which are under the control of non-state actors; as a result the SIAs and subsequent PCCS did not occur in these districts. In contrast, all districts within Puntland and Somaliland states, as well as those in the Benadir administration, were accessible.

Interviews were conducted with mothers and caregivers who were over the age of 15 and resided in the selected clusters, provided they consented to participate in the survey. Mothers or caregivers who were under the age of 15, as well as those who did not provide consent, were excluded from participation in the survey.

### Survey tools

A structured questionnaire was developed to systematically gather data on geographic, demographic, and socio-contextual factors, alongside communication practices pertaining to immunization. This instrument was adapted from a previously utilized questionnaire for Post-Campaign Coverage Surveys (PCCS) in Somalia, with additional questions incorporated as suggested by a WHO expert group [12]. Overall, we adhered to the WHO guideline on coverage survey indicators [15]. A preliminary draft was disseminated to WHO, the Ministry of Health (MoH) and UNICEF for expert review, with modifications made based on the recommendations received. To enhance comprehension and accessibility for respondents, the finalized English version of the instrument was translated into Somali and uploaded it into the KoboCollect app, making it accessible to enumerators via their Android mobile devices or computers.

### Sample size Estimation

As shown in Table 1, we followed the WHO-2018 recommended vaccination coverage cluster surveys sample size calculation [15].

**Table 1:** Sample size calculation

Indicators	Parameters used for the calculation
**Number of strata = 7**	Coverage estimates of SIA campaign
**Expected coverage falls 50%−70%**	Number of respondents required to estimate the coverage for a simple random sample to be done. This number is derived with assumption that expected coverage of 50%−70 and with desired precision of 5% (WHO manual 2018)
**Desired precision = 5%**	To inflate the number respondents to achieve same level precision, taking 12 as target number of respondents per cluster, ICC of 0.167 and adjusting the variation occurred due to survey weight as 0.5
**Effective sample size = 401**	
**Design effect = 4**	
**Average number of households to find an eligible child = 1**	Three parameters, crude birth rate, infant mortality rate and household size from different literature (DHS-2020, World bank, and UNICEF)
**Non-response rate = 1.11**	As there is no similar survey available, we used estimation of 10%
**Total number of completed interviews = 11,228**	Multiplication of three parameters; number of strata, effective sample size, and design effect
**Total number of HH to visit = 12,463**	Multiplication of total number of completed interviews required, average number of HH to visit to get an eligible child, and non-response rate
**Total number of HH to visit per stratum = 1780**	Multiplication of sample size, design effect and average number of HH to visit to get eligible child and total number of completed interviews required
**Total number of clusters per stratum = 134**	Three parameters were used: Effective sample size, design effect and target number of respondents
**Total number of HH per cluster = 14**	It is the outcome of average number of HH to visit to get eligible child, non-response rate and target number of respondents.
**Total number of clusters = 938**	This is the product of total number of clusters per stratum and the number of strata

### Sampling procedure

The survey used a two-stage cluster sampling methodology, aligning with WHO guidelines for post-campaign coverage evaluation [15]. As the first stage of selection, a total of 938 EAs were randomly selected from a sampling frame nationwide. Afterwards, about 134 enumeration areas (EAs) were selected randomly from each state. For the second stage of selection, a simple random sampling method was employed to choose households within each enumeration area\cluster. Prior to the official commencement of the survey, we conducted 287 pilot interviews to evaluate the questionnaire’s structure, response validity, and technical functionalities, including skip logic and filter mechanisms. This process facilitated refinement prior to finalization. The pilot study also enabled testing of the cluster maps and the identification and resolution of any issues with the fieldwork, thereby ensuring the integrity and accuracy of the survey data.

For the randomization of both clusters and households, an Excel spreadsheet was utilized. The serial numbers of clusters within each stratum were included in the spreadsheet, and Excel was then used to randomly generate selections of clusters. Similar procedure was employed to randomly select households from each cluster.

### Data collection

We organized a team comprising two enumerators and a supervisor to visit the selected clusters within each district two days before the survey began. To oversee data collection across all regions, we employed a ‘live data collection monitoring’ system using KoboCollect geocoordinates (GPS). This system captured data for all sampled settlements and households, ensuring location verification and aiding in data validation. It allowed the survey supervisory team to promptly address any feedback regarding data quality or completeness and take immediate corrective actions as needed to maintain the survey’s integrity. All eligible children within the household were included in the data collection. For each child, fingermarks were inspected, vaccination cards were reviewed and photographed, and parents’ reports were recorded. Consequently, the SIAs coverage results were determined using one or a combination of the following methods: verbal reports, card evidence, and fingermark verification. Additionally, parents were asked on whether their child had received the measles vaccine prior to the current campaign. The calculation of measles vaccination rates preceding the SIAs was based on the verbal responses obtained from parents.

### Data management

The data collection and processing occurred concurrently. We utilized the KoboCollect app for data collection, allowing field teams to immediately input and transfer information. Our technical team carefully monitored the data collection process. The use of the KoboCollect app for data collection has helped eliminate human errors by ensuring that enumerators cannot leave any fields blank during interviews. As a result, the dataset contains almost no missing variables. Any discrepancies or errors were promptly addressed by informing the district coordinator, who facilitated necessary corrections. The study team conducted a thorough review of each dataset received, providing feedback and resolving any inconsistencies directly with the field teams. We ensured that the household number, cluster identification code, district and state are all correctly included in the tool. The data manager collaborated with the statistician to clean the dataset by implementing a series of checks for each variable. We utilized frequency and cross-tabulation, alongside descriptive statistics, to identify any outliers.

### Data analysis

The detailed results of the sample data (unweighted) and the overall nationwide coverage assessments were thoroughly analyzed. We calculated vaccine coverage during supplementary immunization activities (SIA) and prior to SIA (which means routine immunization). To calculate the national coverage, a survey weighting methodology was applied. Specific procedures were used to determine cluster and household weights, following the formula outlined in the WHO Vaccination Coverage Cluster Survey 2018 [15].

In the analysis phase, both descriptive and inferential statistics were applied. Descriptive statistics, including frequency counts and proportions, enabled us to summarize the general attributes of the sample. Conversely, inferential statistics were used to estimate national vaccine coverage, featuring point estimates and confidence intervals (Wilson 95% Confidence Intervals), with the application of survey weights.

### Calculation of survey weight

The design weights were computed for every cluster and household selected to participate in the survey. The design weight is the inverse of probability of selecting a household to be interviewed.

We calculated the design weight using the following formula:

Household weight for household $\:i=\:\frac{1}{Probability\:household\:i\:was\:selected\:into\:the\:samples}\:$.

The sample was selected through a two-stage process; therefore, the overall probability of household $\:i$ will be the product of the selection probabilities at each stage.

In the first stage, selection of 134 PSUs from every state (stratum).

The probability of selecting $\:{PSU}_{i}$ in stratum ℎ is:

$$\:{P}_{1}=\:\frac{{PSU}_{h}}{{\sum\:PSU}_{h}}$$

where $\:{PSU}_{h}$ is the PSUs to be selected in stratum h and $\:{\sum\:PSU}_{h}$ is total number of PSUs in stratum h.

In the second stage, 14 households (SSUs) were selected from each sampled cluster.

The probability of selecting SSUi from PSU*i* in stratum ℎ is:

$$\:{P}_{2}=\:\frac{q}{m}$$

where $\:q$ is the fixed number of households in $\:{PSU}_{i}$ in stratum h and $\:m$ is the total number of households in $\:{PSU}_{i}$ in stratum h. Therefore, the household weight will be:

$$\:Household\:weight=\:\frac{1}{{P}_{1}*{P}_{2}}$$

During the analysis, we made careful consideration of the following:

- **Sample Description**: Unweighted data was utilized to describe key demographics, including gender and literacy level, decision-making processes regarding vaccination, and reasons for not receiving the measles vaccines.
- **Overall Coverage**: Weighted data was used to assess the overall coverage of measles vaccination, calculated with Wilson 95% confidence intervals.
- **Demographic Analysis**: Coverage was further broken down (with weighted Wilson 95% confidence intervals) by settlement.
- **Zero-Measles Dose Analysis**: The percentage of children who still had a zero-measles dose despite the Supplemental Immunization Activities (SIA) was calculated with weighted Wilson 95% confidence intervals.

### Ethical considerations

As human subjects involved in this study, ethical principles were strictly considered. according to the Helsinki Declaration of Research Ethics, which states: “The well-being of the individual research subject must take precedence over all other interests”. The crucial ethical considerations include full information disclosure about the research project to the study participants, i.e. the aims and methods of the study, the source of funding, any possible conflicts of interest, anticipated advantages and possible disadvantages of the study, the time required for participation and any possible discomfort that the research project might entail. Accordingly, we explained to the participants about the study’s objectives, as well as the importance of the study and any disadvantages that may be associated with participation in the study such as the time to spend the interviews and filling out questionnaires. Because survey participation must be exclusively voluntary, freedom to participate were given to participants without any coercion, while the confidentiality of their information was assured to them. Lastly, after they fully understood what is taking place in the study, informed oral consent was obtained from each participant. In addition, the research protocol was approved by ethical committee of the Somali National University (Number: JUS/KCC&CT/2011/2025). We ensured the removal of all personally identifiable information by anonymizing the data. Pictures of vaccination cards were deleted from personal devices and saved on institution-owned, password-protected devices accessible only to the study team. These images were deleted after data was fully anonymized.

## Results

### Description of the sample

We conducted interviews with 12,832 families, living in 938 clusters, covering 17,700 children across seven states in the country. Among the children, 46.46% were girls and 53.54% were boys. In terms of literacy levels, 51.49% of caretakers could read and write, while the remaining participants were illiterate. Most children (56.24%) lived in urban areas, followed by 25.77% who lived in rural areas and 14.24% in nomadic areas. Additionally, 3.76% of children lived in Internally Displaced Persons (IDP) camps.

### Coverage

Overall, the national estimates show that 73% of children across the seven states received the measles vaccine during SIAs, with a 95% confidence interval (CI) of 68.14% to 77.26%. Vaccination coverage varied significantly between states. Somaliland and Puntland had the highest coverage rates at 87.4% (CI: 81.7–91.3) and 87.1% (CI: 82.7–90.5), respectively. Conversely, the lowest coverage was observed in Hirshabelle at 48% (CI: 32.6–63.8) and Southwest states at 50% (CI: 40.4–59.7) (Table 2). There were 75 clusters with no single child received the vaccination during SIAs. Of these, 50 clusters, which account for 67%, are located either in Lower Shabelle region in Southwest State (25 clusters) or in the Hiran region in Hirshabelle State (25 clusters).

**Table 2:** Measles vaccination coverage (weighted)

States	Vaccination coverage (%)	95% CI	SE	Unweighted *N*	Weighted *N*
Somalia 2025	72,9	(68.14, 77.26)	2,3	13,687	13,064
Banadir	76,4	(67.8–83.2)	4,0	2,066	952
Galmudug	78,2	(57.6–90.5)	8,8	1,853	806
Hirshabelle	48,0	(32.6–63.8)	8,4	1,441	957
Jubbaland	81,9	(73.5–88.0)	3,7	2,004	1,950
Puntland	87,1	(82.7–90.5)	2,0	2,365	983
Somaliland	87,4	(81.7–91.3)	2,4	2,184	5,496
Southwest	50,0	(40.4–59.7)	5,0	1,774	1,920

Table 3 shows the national measles vaccination coverage is lowest among nomadic populations (71%, CI, 57.78–81.3). There is a slight difference in vaccine coverage between males and females, with 74.3% (95% CI: 69.62–78.44) of males vaccinated compared to 71.4% (95% CI: 65.75–76.44) of females.

**Table 3:** Measles vaccine coverage by geography, age category and sex (weighted)

Background characteristics	Children receiving measles vaccine	
	%	95% CI
**Geographic**		
ID/refugee	76.6	57.70–88.70
Nomadic	70.9	57.78–81.30
Rural	75.2	64.02–83.72
Urban	72.5	66.33–77.91
**Sex**		
Male	74.3	69.62–78.44
Female	71.4	65.75–76.44
**Age group**		
6–23	73.8	68.75–78.32
24–35	72.0	65.91–77.45
36–59	72.7	67.15–77.69

The national coverage of measles vaccination prior to SIAs was 78.6% (CI, 74.6–82.09). Puntland and Benadir states had the highest coverage, with rates of 89% (CI: 85.2–92.0) and 84% (CI: 79.9–87.0) respectively. In contrast, the lowest coverage rates were observed in Hirshebelle at 71% (CI: 55.1–82.9) and Southwest states at 72% (CI: 63.7–78.3), (Table 4).

**Table 4:** Measles vaccine coverage prior to the SIAs (weighted)

States	(%)	95% CI (%)	SE	Weighted *N*
Somalia	78,6	(74.6 −82.09)	**1,9**	14,073
Banadir	83,8	(79.9–87.0)	1,7	1,043
Galmudug	83,2	(76.2–89.9)	3,5	868
Hirshabelle	71,0	(55.1–82.9)	7,4	1,414
Jubbaland	81,4	(72.9–87.7)	3,8	1,939
Puntland	89,1	(85.2–92.0)	1,7	1,006
Somaliland	80,4	(71.5–87.0)	4,0	5,057
Southwest	71,6	(63.7–78.3)	3,8	2,746

As shown in Table 5, the percentage of zero-measles dose children even after the SIAs was 12.9% (CI: 10.17–16.23). About 24.5% of these children were found in Hirshebelle, and in Southwest with a rate of 23.6%. By settlement, the highest proportion of zero-doses children were found among nomads (15.4%). Conversely, the highest proportion of children with two measles doses were found in Puntland (80%, CI: 75.34–83.98) followed by Somaliland (76%, CI: 66.48–82.75).

**Table 5:** Measles vaccination rates before and during SIAs by dose, stratified by state and settlement type (weighted)

Background characteristics	Zero dose		One dose		Two doses	
	%	95%CI	%	95%CI	%	95%CI
**Somalia**	12.9	10.17–16.23	22.7	19.33–26.42	64.4	59.68–68.89
**State**						
Benadir	11.2	8.70 −14.29	17.5	11.92–24.86	71.3	63.21–78.30
Galmudug	7.3	4.34–12.09	22.9	10.08–44.03	69.8	50.86–83.74
Hirshabelle	24.5	12.45–42.68	31.9	22.15–43.54	43.5	28.91–59.40
Jubaland	7.01	4.83–10.23	22.6	15.04–32.55	70.3	59.48–79.26
Puntland	3.8	1.99–7.15	16.2	12.44–20.78	80.0	75.34–83.98
Somaliland	7.8	4.52–13.08	16.7	11.04–24.45	75.5	66.48–82.75
Southwest	23.6	17.12–31.50	31.3	23.94–39.74	45.1	36.10 −54.52
**Geography**						
ID/refugee	9.1	5.31–15.15	34.3	20.03–52.23	56.5	39.03–72.56
Nomadic	15.4	8.96–25.21	23.4	13.71–36.97	61.2	46.11–74.40
Rural	10.7	6.33–17.52	24.9	19.12–31.68	64.4	56.42–71.70
Urban	13.3	9.85–17.70	21.4	17.36–26.12	65.1	59.19–70.93

**Table 6:** Measles SIAs coverage among previously zero-dose children

Previous measles vaccination	SIAs measles vaccination	
	No	Yes
No	60.2% (49.92–69.68)	39.78% (CI: 30.32–50.08)
Yes	18.0 (14.37–22.35)	81.98% (CI: 77.65–85.63)

The Table 6 shows weighted national estimates about SIAs coverage among children previously never vaccinated against measles vs. those who had previously received one or more doses. The SIAs coverage was 39.8% (CI: 30.32–50.08) among children with no prior measles vaccination (zero-dose), and 82% (CI: 77.65–85.63) among those who had previously received at least one dose

**Fig. 1:**
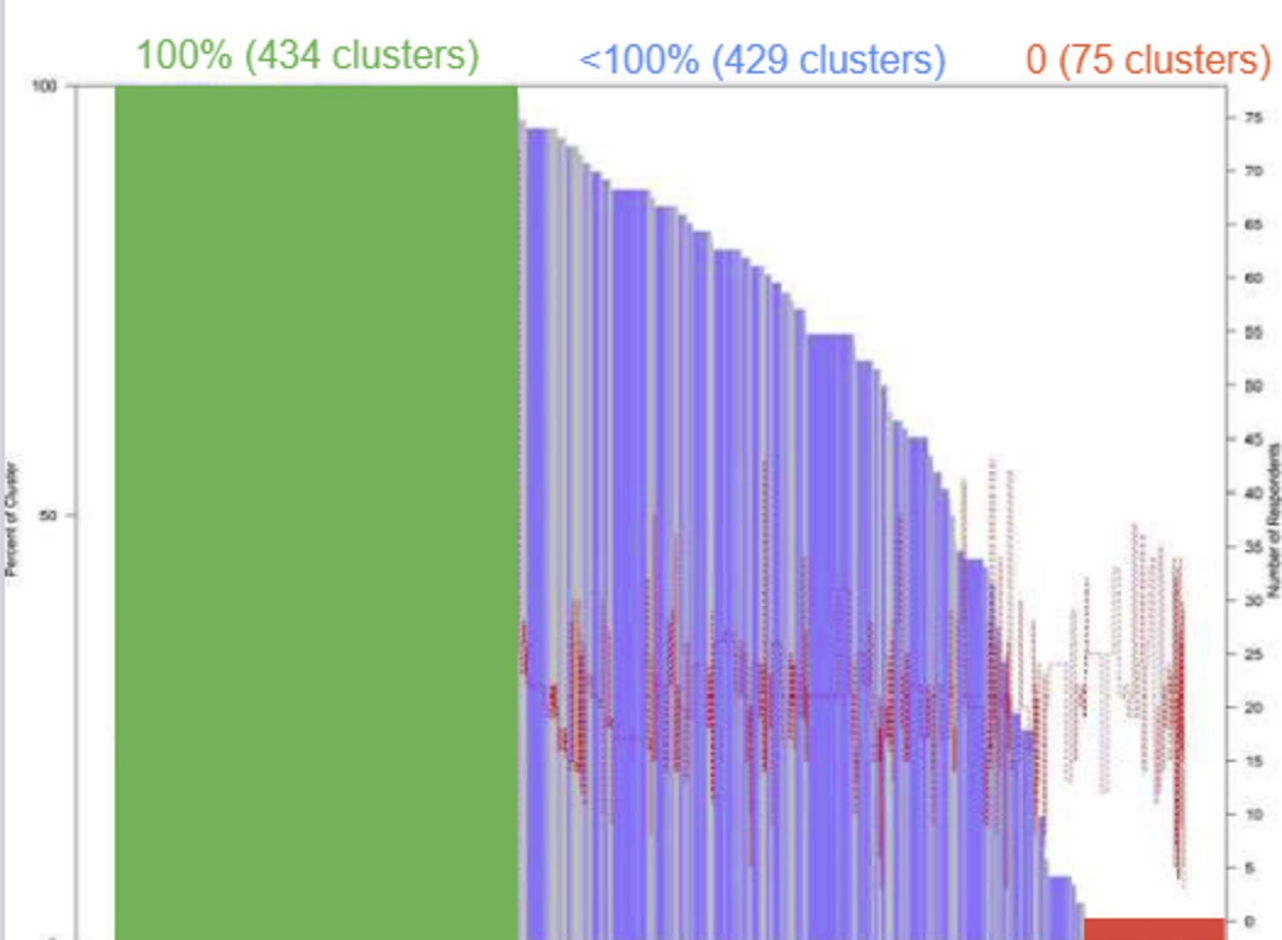
Organ pipe plot of proportion of children vaccinated per cluster in 6 states in Somalia and Somaliland. The organ pipe plot illustrates the proportion of children in each surveyed cluster who received the measles vaccine dose. Each vertical bar represents a cluster, with the bar’s width proportional to the sum of the survey weights from that cluster. The height of the bar indicates the proportion of children who received the measles vaccine dose. Clusters are arranged from left to right in order of coverage, from high to low. The green represents clusters with 100% of children being vaccinated, blue\gold represent clusters that < 100 children is vaccinated, the red color represent cluster that no child was vaccinated. Thin red line runs through the figure, representing the number of children sampled in each cluster. The bar heights should be interpreted using the scale on the left, while the number of children in each cluster can be read from the scale on the right

As demonstrated in the organ pipe plot, among the 17,700 children surveyed across 938 clusters, 436 clusters attained full vaccine coverage in SIAs. In contrast, 75 clusters reported zero coverage (Fig. 1).

It’s noteworthy that of the 75 clusters with zero coverage in the SIAs, nearly 70% are concentrated in the Hiran region of Hirshabelle State and the Lower Shabelle region of South-West State (Table 7).

**Table 7:** Clusters with zero measles SIA coverage by region

Region	Freq.	Percent	Cum.
Banadir	2	2.7	2.7
Bay	7	9.3	12.0
Galgadud	2	2.7	14.7
Gedo	2	2.7	17.3
Hiran	25	33.3	50.7
Lower Shabelle	25	33.3	84.0
Middle Shabelle	3	4.00	88.00
North Mudug	1	1.33	89.33
Nugal	2	2.67	92.00
South Mudug	6	8.00	100.00
Total	75	100.00	

The reasons for not being vaccinated varied among the states. In six states, the primary reason was a “lack of awareness about the vaccination campaign (SIAs).” In contrast, in Somaliland, the two main reasons were either the “absence of a guardian” or “the child being unwell at the time of vaccination” (see Table 8).

**Table 8:** Reasons for not vaccinating children by state (%) (measles) (unweighted)

Reason for Not Vaccinated	Benadir	Galmudug	Hirshabelle	Jubaland	Puntland	Somaliland	Southwest	National
Didn’t Know About the Campaign	17%	20%	31%	36%	36%	13%	19%	24%
Other	8%	12%	12%	16%	10%	6%	26%	16%
Missing Vaccinator at the Site	10%	10%	23%	2%	1%	4%	20%	15%
Site of Vaccination Was Not Known	2%	2%	1%	1%	1%	6%	21%	8%
Vaccine Not Available at the Site	4%	8%	19%	1%	4%	4%	0%	7%
Fear of Side Effects	11%	12%	4%	9%	8%	3%	2%	6%
Parent/Guardian Was Missing	8%	13%	0%	12%	9%	14%	2%	6%
Child Already Received Measles Vaccine	16%	3%	5%	6%	11%	7%	0%	5%
Site of Vaccination Too Far	1%	6%	1%	3%	2%	4%	4%	3%
Child Was Sick at Time of Vaccination	5%	3%	0%	3%	8%	14%	1%	3%
Not Authorized by Head of Household	5%	4%	1%	1%	2%	3%	0%	2%
Fear of Injections	4%	1%	0%	2%	2%	0%	1%	1%
Too Busy to Take a Child	1%	1%	0%	1%	3%	7%	1%	1%
Lack of Confidence in the Vaccine	4%	2%	0%	1%	0%	2%	1%	1%
Absent or Travelling During Campaign	1%	1%	1%	2%	1%	3%	0%	1%
Confused with Other Vaccines	2%	0%	0%	3%	1%	2%	0%	1%
Vaccination Unsuitable	1%	0%	0%	1%	0%	3%	0%	0%

The denominator is the total number of non-vaccinated in each region

As shown in Table 9, ‘community health workers’ were the leading source of vaccination information nationally, accounting for 36% of responses, followed by ‘social mobilizers’ at 23%.

**Table 9:** Source of information about the campaign by state (%) (unweighted)

Source of Information	Benadir	Galmudug	Hirshabelle	Jubaland	Puntland	Somaliland	Southwest	National
Community Health Workers	24%	37%	24%	39%	48%	41%	37%	36%
Family	4%	2%	0%	4%	3%	1%	4%	3%
Friend	4%	4%	7%	6%	8%	3%	5%	5%
Internet	19%	5%	9%	5%	3%	19%	3%	9%
Mosque Announcement	0%	0%	0%	0%	0%	0%	2%	0%
Other	0%	0%	0%	0%	0%	1%	0%	0%
Radio	16%	8%	15%	15%	3%	8%	9%	11%
School	1%	1%	1%	2%	1%	2%	1%	1%
Social Mobilisers	15%	34%	32%	15%	30%	16%	27%	23%
Television	15%	2%	4%	3%	2%	8%	5%	6%
Village Chief	1%	7%	8%	11%	2%	1%	8%	5%

## Discussions

The routine immunization coverage for children is often enhanced by supplementing it with a second dose opportunity for measles vaccine through supplemental immunization activities (SIAs) in high measles burden countries. The Somalia’s nationwide measles vaccine coverage during the Supplemental Immunization Activity in 2025 (SIAs-2025) was found to be 73%. However, this overall figure conceals considerable state disparities. Regions affected by recent conflicts [13] typically exhibit lower vaccination rates, with the most deficient coverage in states experiencing recent active conflict. The study further reveals that zero-dose children in Somalia are predominantly located in the conflict-afflicted regions of Hiran and Lower Shabelle. Conversely, the highest measles vaccination coverage is observed in the more stable regions of Somaliland and Puntland. This finding concords with global trend that conflict-affected areas bear a large share of polio and measles cases—nearly 70% of global polio cases (2010–2016) occurred in such regions [7], and two-thirds of unimmunized children live in unstable countries [16]. Conflict and instability disrupt healthcare infrastructure and services, making it difficult for vaccination programs to reach these areas [17]. In conflict-affected areas in Somalia, logistical challenges, safety concerns, and population displacement may impede access to vaccination services. This not only diminishes the national vaccination coverage, but also exacerbates significant disparities in vaccination coverage among children in the country. As a result, children residing in these regions may be at an increased risk of measles exposure, potentially serving as reservoirs for the disease and contributing to recurring outbreaks nationwide.

There are 75 clusters that were not reached by the Supplementary Immunization Activities (SIAs). The vast majority of these clusters are located in states that experience frequent conflicts [13]. This clearly demonstrates the influence of conflict on measles vaccination efforts, with the potential to deprive children in conflict-affected areas of immunization. Previous studies, such as those by UNICEF [18], have documented similar patterns where conflict significantly disrupts healthcare delivery and vaccination initiatives [19]. These disruptions not only hinder immediate access to essential vaccines but also contribute to longer-term public health challenges in maintaining herd immunity and preventing outbreaks [20]. According to UNICEF, while measles mortality is typically below 1% in stable regions, it can rise to 30% among children in conflict regions [21]. The low vaccination coverage rates in certain states in Somalia, which are prone to conflict, as well as in pastoral communities, undermine the overall national vaccination performance.

Prior study shows that conducting regular SIAs with high coverage rates is an effective method to avert measles outbreaks [22]. Our study revealed that approximately 40% of children who previously had not received any vaccine doses were reached during the 2025 SIAs in Somalia. While this is an encouraging development, indicating progress in expanding immunization coverage, there remains significant room for improvement. This achievement underscores the effectiveness of SIAs in bridging gaps in routine immunization efforts but also highlights the need for targeted strategies to reach the remaining unvaccinated child population in areas affected by conflicts and marginalized communities such as pastoralists. Enhanced outreach, focused interventions in conflict-ridden states and vulnerable communities, and continuous monitoring and evaluation are essential to further increase vaccine coverage and ensure all children receive measles immunizations in Somalia.

One of the primary reasons for not vaccinating children in Somalia during SIAs was a lack of awareness about the vaccination campaigns. This constitutes a significant gap, underscoring the necessity for a well-coordinated national community sensitization or awareness campaign prior to SIAs. Such a campaign should aim to educate families about upcoming vaccination drives and emphasize the benefits of vaccinating their children. Sensitization and awareness campaigns implemented prior to SIAs have the potential to significantly increase vaccine demand by educating communities on the importance of immunization, addressing vaccine hesitancy, and mobilizing community support, thereby enhancing participation rates and ultimately improving vaccination coverage [23].

Our study found that community health workers are the main source of vaccine information in Somalia. Community Health Workers (CHWs), as essential frontline personnel, are vital to routine vaccination efforts [24]. They are instrumental in creating demand for childhood vaccinations, linking community members with the formal healthcare system, and engaging extensively within communities, schools, and religious centers [24]. Prior research highlighted the significant impact CHWs have on increasing demand for vaccinations in countries such as Nepal, Senegal, and Zambia [25]. Community Health Workers (CHWs) were more active in delivering vaccine information in states with high vaccination rates, such as Puntland (48%) and Somaliland (41%). This shows clearly the potential of community health workers in delivering vaccination information to populations including nomadic communities. Strengthening community health workers involvement in vaccination awareness in all regions may increase the vaccine information, demand and vaccine acceptance in Somalia.

The study acknowledges some limitations. First, the sampling framework was based on outdated population estimates. While these estimates are regularly updated to reflect demographic changes, they are inherently susceptible to inaccuracies due to the dynamic nature of population growth, migration, and mortality rates. Inaccuracies in these estimates can lead to sampling bias, possibly reducing the precision of population coverage estimates. Secondly, the exclusion of 15 districts, which are under the control of non-state actors, introduces a geographic and socio-political limitation to the study’s applicability. These areas might differ significantly from government-controlled regions. The omission of these districts means the study’s findings cannot be extrapolated to cover these areas, limiting the generalizability of its conclusions.

### Recommendations

To address the weaknesses identified in the SIAs in Somalia and enhance vaccine coverage, the following recommendations should be considered.

1. Develop Targeted Outreach Programs: Design initiatives specifically aimed at reaching zero-dose children and overlooked clusters to ensure every child in country receives necessary vaccinations.
2. Enhance Training for Community Health Workers: Provide community health workers across the country with extensive training and resources, empowering them with in-depth knowledge and effective communication skills to promote vaccinations effectively.
3. Launch a Comprehensive Sensitization Campaign: Prior to SIAs, initiate a nationwide awareness effort using various channels, CHW, and local influencers to engage diverse populations and boost the visibility of vaccination efforts.

## Data Availability

Data is provided within the manuscript.
